# *VDR* rs2228570 Polymorphism Is Related to Non-Progression to AIDS in Antiretroviral Therapy Naïve HIV-Infected Patients

**DOI:** 10.3390/jcm8030311

**Published:** 2019-03-05

**Authors:** María A. Jiménez-Sousa, José Luis Jiménez, Amanda Fernández-Rodríguez, Oscar Brochado-Kith, José María Bellón, Félix Gutierrez, Cristina Díez, Enrique Bernal-Morell, Pompeyo Viciana, María A. Muñoz-Fernández, Salvador Resino

**Affiliations:** 1 Unidad de Infección Viral e Inmunidad, Centro Nacional de Microbiología, Instituto de Salud Carlos III, Carretera Majadahonda-Pozuelo, Km 2.2, 28220 Madrid, Spain; amandafr@isciii.es (A.F.-R.); brochado1993@gmail.com (O.B.-K.); 2 Plataforma de Laboratorio, Hospital General Universitario “Gregorio Marañón”, 28007 Madrid, Spain; joseluis.jimenez@salud.madrid.org; 3 Fundación para la Investigación Biomédica, Hospital General Universitario Gregorio Marañón, Instituto de Investigación Sanitaria Gregorio Marañón (IiSGM), 28007 Madrid, Spain; bellon23@gmail.com; 4 Unidad de Enfermedades Infecciosas, Hospital General de Elche & Universidad Miguel Hernández, 03202 Alicante, Spain; gutierrezfel@gmail.com; 5 Servicio Microbiología, Unidad de Enfermedades Infecciosas/VIH, Hospital General Universitario Gregorio Marañon, 28007 Madrid, Spain; crispu82@gmail.com; 6 Servicio de Enfermedades Infecciosas, Hospital General Universitario Reina Sofia, 30003 Madrid, Spain; ebm.hgurs@gmail.com; 7 Servicio de Enfermedades Infecciosas, Hospital Virgen del Rocío, 41013 Seville, Spain; pompeyo.viciana.sspa@juntadeandalucia.es; 8 Sección Inmunología, Laboratorio Inmuno Biología Molecular, Hospital General Universitario Gregorio Marañón, IiSGM, and Spanish HIV HGM BioBank, 28007 Madrid, Spain; mmunoz.hgugm@gmail.com; 9 Networking Research Center on Bioengineering, Biomaterials and Nanomedicine (CIBER-BBN), 28007 Madrid, Spain

**Keywords:** single nucleotide polymorphisms, *VDR*, LTNPs, AIDS, non-progression

## Abstract

Background: Vitamin D is a fundamental regulator of host defenses by activating genes related to innate and adaptive immunity. In this study, we analyzed the association among single nucleotide polymorphisms (SNPs) in the *vitamin D receptor* (*VDR*) gene, with clinical patterns of AIDS progression in antiretroviral treatment (ART)-naïve HIV-infected patients. Methods: We conducted a retrospective study in 667 HIV-infected patients, who were classified within three groups according to their AIDS progression pattern (183 long-term non-progressors (LTNPs), 334 moderate progressors (MPs), and 150 rapid progressors (RPs)). Five *VDR* SNPs (rs11568820, rs4516035, rs2228570, rs1544410, and rs7975232) were genotyped using Agena Bioscience’s MassARRAY platform. Results: Significant association results were found for rs2228570. Within all HIV patients, the presence of T allele at *VDR* rs2228570 SNP was protective against AIDS progression (ordinal outcome) under additive (adjusted odds ratio (aOR) = 0.75; *p* = 0.009), dominant (aOR = 0.69; *p* = 0.015), and codominant (aOR = 0.56; *p* = 0.017) inheritance models. In addition, the same allele was protective under additive and codominant inheritance models when we compared with LTNPs vs. RPs [aOR = 0.64 (*p* = 0.019) and aOR = 0.37 (*p* = 0.018), respectively] and when we compared MPs vs. RPs [aOR = 0.72 (*p* = 0.035) and aOR = 0.45 (*p* = 0.028), respectively]. Conclusions: The *VDR* rs2228570 T allele was related to a lower AIDS progression pattern in ART-naïve HIV-infected patients. These findings expand upon the knowledge about HIV pathogenesis in untreated HIV-infected patients with different clinical outcomes.

## 1. Introduction

The natural history of human immunodeficiency virus (HIV) infection has a substantial inter-individual variability on the progression of acquired immunodeficiency syndrome (AIDS) among antiretroviral treatment (ART)-naïve, HIV-infected patients [1]. Three major patterns can be identified according to the AIDS progression rate: Long-term non-progressors (LTNPs), moderate progressors (MPs), and rapid progressors (RPs) [2,3]. LTNPs are patients who do not progress to AIDS over an extended period, and they have a total or partial control of HIV replication, high CD4+ lymphocyte numbers, and absence of clinical symptoms. MPs (also known as moderate, intermediate or typical progressors) in opposition to the extremes of the clinical-immunological distribution (LTNPs and RP), are patients without viremia control, who slowly progress to AIDS during an extended period after seroconversion (3 to 10 years after seroconversion). RPs are patients who rapidly progress to AIDS within the first year after seroconversion. This inter-individual variability to AIDS progression is probably the result of a complex interaction between genetic background, immune system, and viral factors [4,5].

Vitamin D (VitD) deficiency is related to osteomalacia, rickets, and non-skeletal diseases, such as infectious diseases [6]. VitD triggers effective anti-microbial pathways against bacterial, fungal, and viral pathogens in host cells, by activating genes that enhance innate and adaptive immunity [7]. Thus, VitD deficiency has been related to a higher incidence and severity of *Mycobacterium tuberculosis*, HIV, and hepatitis C virus (HCV) infection [8,9]. Regarding HIV infection, VitD deficiency is present in 70–85% of patients infected with HIV, and may be related to non-HIV-related risk factors (gender, advanced age, limited sunlight exposure, genetic background, among others) and HIV-related factors (HIV infection itself, ART, and higher incidence of malnutrition and comorbidities, among others) [9,10]. High levels of VitD seem to provide a natural resistance to HIV infection, whereas low levels of VitD are related to a higher values of HIV viral load in plasma, inflammation, immune activation, decrease of CD4+ T-cells and rapid AIDS progression [7]. Moreover, VitD levels are related to CD4+ T-cell recovery after 24 weeks of VitD supplementation, whereas VitD deficiency is associated with impaired CD4+ T-cell count recovery in HIV-positive patients on ART [7].

VitD effects are mediated by its nuclear receptor, vitamin D receptor (VDR), which acts as a transcription factor that promotes the expression of vitamin D-related genes [11]. Additionally, VitD may bind to an external VDR, which is located on the cell membrane (VDRm), and to promote the secretion of growth factors and cytokines through the modulation of nuclear transcription factors [7]. The *VDR* gene is located at human chromosome 12q13, the VDR region is organized in three main haplotype blocks (A, B and C) and several sub-blocks, which vary in size and distributions among European, African, and Asian populations [12,13]. Some of the most studied single nucleotide polymorphisms (SNPs) have been Bsm1 (rs1544410) and ApaI (rs7975232), located at block B; GATA (rs4516035) and Cdx2 (rs11568820) located at block C2, and the Fok1 (rs2228570), located at exon 2 between blocks B and C. Several single nucleotide polymorphisms (SNPs) at the *VDR* gene have been associated with non-skeletal health problems, especially infectious and auto-immune related diseases [14]. There is scarce information about *VDR* SNPs on the progression to AIDS in naïve HIV-infected patients [15,16,17,18], and these studies were performed with lower sample sizes. Thus, since *VDR* SNPs have been associated with a large number of immune-mediated diseases, we hypothesized that these SNPs could play an important role in the clinical progression of HIV-infected patients, and could be associated with clinical patterns of AIDS progression. 

### Objective

We aimed to analyze the association among five *VDR* SNPs and the clinical patterns of progression, categorized as LTNPs, MPs, and RPs, for the natural history of HIV infection in European ART-naïve HIV-infected patients from two large Spanish cohorts (Cohort of the Spanish AIDS Research Network (CoRIS) and Cohort of LTNPs).

## 2. Patients and Methods

### 2.1. Patients

We conducted a retrospective study in 667 European ART-naïve HIV-infected patients from both CoRIS and LTNPs cohorts, whose samples were available at the Spanish HIV HGM Biobank [19]. The methodological and organizational aspects, and the characteristics of the subjects enrolled in the present study, have been previously described [20]. Moreover, 113 healthy blood donors (HIV, HCV, and HBV negative subjects) from “Centro de Transfusión de la Comunidad de Madrid” were used as a Control-group. The programs were approved by the institutional review boards of each participating center. In addition, the Research Ethics Committee of the Instituto de Salud Carlos III (ISCIII) approved this study (CEI PI_2010-v3), which was conducted under the ethical principles of the Declaration of Helsinki. All patients signed an informed consent form.

Patients infected with HIV were classified within three groups depending on their drop in CD4 T-cell counts and AIDS disease progression rates in the absence of ART [21,22]: (a) 183 LTNPs, who had asymptomatic HIV infection over ten years after seroconversion, and always showing CD4+ ≥500 cells/mm^3^ and RNA-viral load ≤10,000 copies/mL. (b) 334 MPs who had an average decrease of 50–100 CD4+/mm^3^ per year for at least two years after HIV diagnosis. (c) 150 RPs, who had two or more CD4+ T-cell values below 350/mm^3^ (recommended threshold for initiating ART) and/or who had AIDS or AIDS-related death within three years after HIV seroconversion (seroincidents). These are three well-defined groups with available sample in the Spanish HIV Biobank, which show the three main patterns of progression to AIDS. All patients had a regular follow up of CD4+ counts and plasma HIV-RNA during the study period.

### 2.2. DNA Genotyping

We selected five *VDR* SNPs, which had been previously related to the risk of acquisition and progression of HIV infection [16,17,18,23,24]. These SNPs are located upstream (rs11568820 and rs4516035), within the coding region (rs2228570 [missense variant]), and at two intronic region (rs1544410 and rs7975232). 

Samples from patients were kindly provided by the HIV BioBank integrated into the Spanish AIDS Research Network (RIS). The samples were immediately processed and frozen after their reception. The total DNA isolation was performed from peripheral blood mononuclear cells with Qiagen columns (QIAamp DNA Blood Midi/Maxi; Qiagen, Hilden, Germany). DNA genotyping was carried out at the Spanish National Genotyping Center (CeGen; http://www.cegen.org/) by Agena Bioscience’s MassARRAY platform (San Diego, CA, USA) with the iPLEX^®^ Gold assay design system.

### 2.3. Statistical Analysis

For the descriptive study, categorical data and proportions were analyzed using the chi-squared test or Fisher’s exact test. Kruskal-Wallis and Mann-Whitney U tests were used to compare continuous variables among independent groups.

The genetic association analysis was carried out according to dominant, recessive, over-dominant, codominant, and additive models. The Mantel-Haenszel linear-by-linear association test (values between −1 and +1), a non-parametric test for measuring the association between ordinal data, was used to explore the distribution of *VDR* genotypes among the three clinical patterns of AIDS progression. We also used Generalized Linear Models (GLMs) adjusted by age at HIV diagnosis, gender, and risk group (men who have sex with men (MSM) versus others). Firstly, a GLM model, with a multinomial distribution (cumlogit-link), was used to evaluate the association of *VDR* SNPs with the three clinical patterns of AIDS progression (ordinal variable: 0, LTNP; 1, MP; 2, RP). The reference category was the LTNP-group. The goodness of fit of each model was evaluated by the Akaike information criterion (AIC) value and Bayesian information criterion (BIC). In order to exclude spurious associations, multiple testing correction was carried out by the false discovery rate (FDR) with the Benjamini and Hochberg procedure. Secondly, a GLM model with a binomial distribution (logit-link) was used for estimating frequency differences of *VDR* SNPs between clinical patterns of AIDS progression (dichotomous variable: LTNPs vs MPs; LTNPs versus RPs; MPs versus RPs). In this case, only SNPs with an adjusted p-value less than 0.05 (after an FDR correction) from the ordinal regression (Step 1) were analyzed. All statistical tests were performed with the Statistical Package for the Social Sciences (SPSS) 22.0 software (IBM Corp., Armonk, CA, USA). Statistical significance was defined as *p* < 0.05. All *p*-values were two-tailed.

Haploview 4.2 software (MIT/Harvard Broad Institute, Cambridge, MA, USA) was used to determine the Hardy-Weinberg equilibrium (HWE) and pairwise linkage disequilibrium (LD), using the standardized D’ and *r*^2^ values.

## 3. Results

### 3.1. Characteristics of the Study Population

Table 1 shows the epidemiological and clinical characteristics of HIV infected patients (183 LTNPs, 334 MPs, and 150 RPs). LTNP-patients had the highest values of age at the moment of HIV diagnosis, age at the study inclusion, percentage of intravenous drug users (IDU) (*p* < 0.001), and the lowest proportion of male patients (*p* < 0.001). Besides, MP and RP groups were diagnosed with HIV infection in the 2000s and had higher percentages of men and homosexual HIV transmission than LTNP group.

### 3.2. Characteristics of VDR SNPs

**Figure 1:**
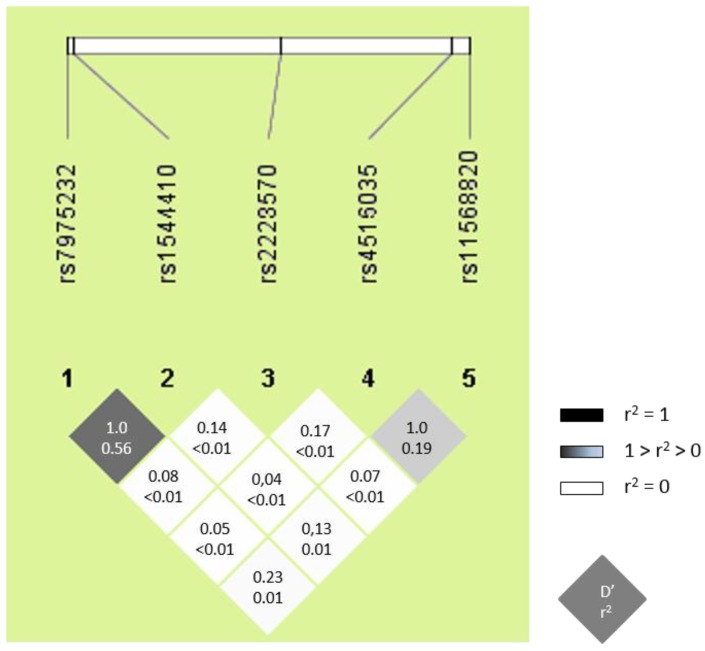
Pairwise linkage disequilibrium (LD) pattern for *VDR* polymorphisms. The locations of each tested SNP, along the genome region, is indicated on top. Each diagonal represents a different SNP, with each square representing a pairwise comparison between two SNPs. The number in each square indicates the magnitude of LD, expressed as D´ and *r*^2^. Color scheme: Grey color intensity decreases with decreasing *R*-squared value. Dark grey indicates strong LD; squares in light grey indicates weaker LD; white indicates very weak or no LD.

All *VDR* SNPs (rs11568820, rs1544410, rs2228570, rs4516035, and rs7975232) were in HWE (*p* > 0.05), the values for the minor allelic frequency (MAF) were higher than 5%, and the genotyping call-rate success was over 95% (Table 2). Similar values of genotypic frequencies for *VDR* SNPs were found in healthy-controls and HIV-infected patients (Table 2). Furthermore, the *VDR* genotype frequencies were in accordance with the NCBI SNP database (http://www.ncbi.nlm.nih.gov/projects/SNP). Moreover, LD values were very high for rs7975232 and rs1544410 (D’ = 1.0), and rs11568820 and rs4516035 (D’ = 1.0); but the *r*^2^ statistic was very low among all *VDR* SNPs (Figure 1), which means that each SNP provides different information.

### 3.3. VDR SNPs and AIDS Progression

**Figure 2:**
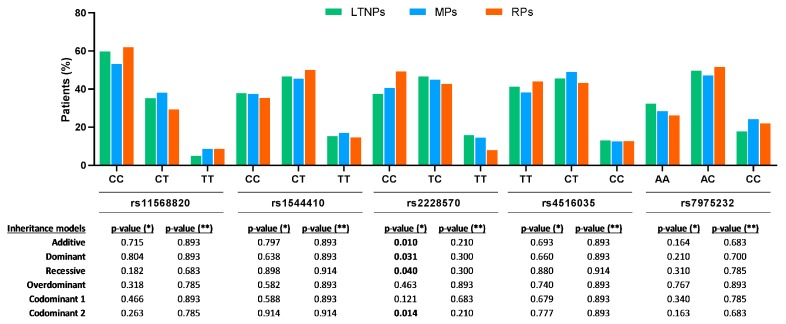
Genetic association of *VDR* polymorphisms, with distinct patterns of AIDS progression in HIV infected patients. *p*-values were calculated by the Mantel-Haenszel linear-by-linear association test. (*), raw *p*-values; (**), *p*-values corrected for multiple testing using the false discovery rate (FDR) with Benjamini and Hochberg procedure (*n* = 30, multiple comparisons). VDR, vitamin D receptor; LTNPs, Long Term Non Progressors; MPs, Moderate Progressor; RPs, Rapid Progressor; Codominant 1, homozygous more frequent versus heterozygous; Codominant 2, more frequent homozygote versus less frequent homozygote.

We explored the genetic association between *VDR* SNPs (rs11568820, rs1544410, rs2228570, rs4516035, and rs7975232) and the three clinical patterns of AIDS progression (LTNP, MP, and RP) by univariate analysis (Figure 2). We found a significant association for only rs2228570 SNP under an additive (*p* = 0.010), dominant (*p* = 0.031), recessive (*p* = 0.040), and codominant (*p* = 0.014) inheritance models. However, after the FDR (Benjamini & Hochberg) controlling procedure, no significant *p*-values were obtained. Despite this, we do not necessarily think that this association between *VDR* rs2228570 SNP, and the clinical patterns of AIDS progression, could be a false positive. Correcting for multiple testing is generally a very strict methodology to maintain the statistical significance for certain SNPs that could be associated with the outcome variable. Furthermore, we also observed that the distribution of patients in each of the three categories of progression to AIDS, according to genotypes, showed a prominent “effect size” of the T allele for rs2228570.

**Figure 3:**
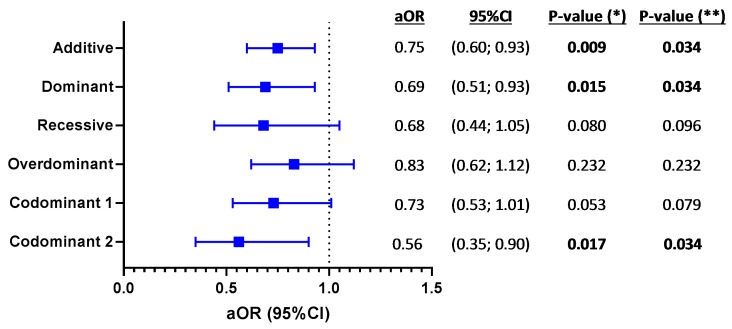
Genetic association of *VDR* rs2228570 polymorphisms with patterns of AIDS progression (ordinal variable) in HIV infected patients. *p*-values were calculated by ordinal regression adjusted for age, gender, and risk category (men who have sex with men (MSM) versus others). (*), raw *p*-values; (**), *p*-values corrected for multiple testing using the false discovery rate (FDR) with Benjamini and Hochberg procedure (*n* = 6, multiple comparisons). VDR, vitamin D receptor; LTNPs, Long Term Non Progressors; MPs, Moderate Progressor; RPs, Rapid Progressor; Codominant 1, codominant 1 refers to heterozygous genotype vs more frequent homozygous (genotype 1); Codominant 2, codominant 2 refers to less frequent homozygote (genotype 2) vs more frequent homozygote (genotype 1).

Afterwards, the GLM tests adjusted for age, gender, and risk category largely confirmed these findings (Figure 3). Therefore, when analyzing all 667 HIV patients, the presence of T allele at *VDR* rs2228570 SNP protected against AIDS progression (ordinal outcome) under additive (adjusted odds ratio (aOR) = 0.75; *p* = 0.009), dominant (aOR = 0.69; *p* = 0.015), and codominant (aOR = 0.56; *p* = 0.017) inheritance models. In this case, *p*-values were also corrected by multiple comparison (FDR -Benjamini & Hochberg), and remained significant.

## 4. Discussion

In this study, we evaluated the distribution of *VDR* SNPs (rs11568820, rs1544410, rs2228570, rs4516035, and rs7975232) among European ART-naïve HIV-infected patients, from two large Spanish cohorts (CoRIS and LTNPs cohorts), grouped by their clinical progression pattern (LTNPs, MPs, RPs). Our most significant finding was that, HIV-infected patients carrying the *VDR* rs2228570 T allele, had lower odds of progression to AIDS.

VitD induces an antiviral effect in the body, that promotes the expression of antiviral genes, reduces the expression of CCR5 on CD4+ T-cells, promotes an HIV-1-restrictive CD38+HLA-DR+ phenotype, and reduces the ability of TNFα to upregulate HIV replication in latently infected CD4+ T-cells [25,26]. The variability that the genetic background of patients may confer differential susceptibility to HIV infection and progression to AIDS [5]. The five *VDR* SNPs analyzed in our study (rs11568820, rs1544410, rs2228570, rs4516035, and rs7975232) were previously involved in protection against HIV infection [18,23,24]. Four of them (rs11568820, rs1544410, rs2228570, and rs4516035) have been related to the AIDS progression in Spanish and Indian cohorts [15,16,17,18]. However, we have only confirmed the significant association of rs2228570 with AIDS progression, in accordance with the findings of Nieto et al. [17] and Laplana et al. [16]. In contrast, Barber et al. [15] and Torres et al. [18] did not analyze the *VDR* rs2228570 SNP. Therefore, rs2228570 polymorphism seem to have a relevant impact in the natural history of our ART-naïve HIV-infected patients, although we should not rule out that other SNPs in high LD, with rs2228570, could be the causal polymorphism.

Previous retrospective studies in very similar populations [16,17] found a weak association in the *VDR* rs2228570 polymorphism. Our data has the key advantage of a much larger sample size and confirms the locus, although the most significant associations came from a slightly different genetic model. We therefore believe that the *VDR* rs2228570 polymorphism might be relevant for HIV progression, although the exact model (additive, recessive, dominant, over-dominant or codominant) remains to be determined. Even without a specific model, this study warrants further investigation on the potential role of *VDR* SNPs, and this one in particular as a partial determinant of HIV progression.

The rs2228570 C->T polymorphism (also called FokI because may be detected by Restriction Fragment Length Polymorphism (RFLP) using the endonuclease FokI is located in the 5′ untranslated region (UTR), three codons before the start codon (ATG) [27,28]. Thus, the C allele (F variant) does not originate any new start codon (ATG), whereas the T allele (f variant) creates a new start codon and a VDR protein, with three extra amino acids [28]. However, discrepant results have been published around the putative implication of rs2228570 polymorphism on the *VDR* expression and vitamin D signaling pathway. On the one hand, the T allele has been related to a less efficient interaction between VDR protein and transcription factor IIB (TFIIB) [29], and to a lower *VDR* mRNA copy number [30]. This might be due to the three extra amino acids of the final protein, which could be disrupting a correct interaction. On the other hand, Oskooei et al. [31] described that the TT genotype was associated with a higher *VDR* expression levels in breast cancer patients. Additionally, there are also studies that have described a lack of association between the *VDR* rs2228570 polymorphism and *VDR* expression in immune cells of European, and South African Populations [32,33], respectively. We did not analyze the effect of rs2228570 SNP at the functional level. However, we hypothesize that the effect of the T allele might cause a decrease in the level of expression of *VDR* and the impairment of its function, which would induce the inhibition of HIV replication and block the destruction of CD4+ T cells. 

### Limitations of the Study

Firstly, this work is a retrospective study that included three groups of patients (defined by changes in CD4 T-cell counts and AIDS disease progression rates) with substantial differences in demographic and clinical characteristics. Thus, LTNPs acquired the HIV infection, mainly by IDU, and were diagnosed at the beginning of the 90s; while MPs and RPs were mainly MSM that were diagnosed of HIV infection more recently (2000s), these patients were predominantly men, with a younger age at the time of HIV diagnosis. These differences could introduce some bias in the study. Besides, the sample size was limited in some sub-groups of patients, which may influence the statistical significance level when the effects of the variables studied are not significant. 

Secondly, our study was completely performed in Spanish Caucasians individuals. Thus, since the frequency of the alleles varies among races, an independent study with different ethnic groups would be interesting in clarifying the association between different genetic backgrounds. We should not rule out the influence of other factors, such as the exposure to sunlight, since vitamin D levels depend on the conversion rate of pro-vitamin D3 to pre-vitamin D3 by sunlight, which is affected by seasonal characteristics, latitude, radiation, and urban living. In our study, HIV-infected patients came from all different regions of Spain.

Thirdly, the HCV coinfection data were available for 75% (500/677) of patients, but the data of HBV coinfection, opportunistic infections, and other non-AIDS-related co-morbidities (e.g., cancer, liver disease, renal impairment, diabetes, obesity, endocrine disease, and cardiovascular disease) were not available. Thus, these co-factors could not be included in the multivariate analysis to account for these effects.

## 5. Conclusions

In conclusion, the *VDR* rs2228570 T allele was related to a lower AIDS progression rate in European ART-naïve HIV-infected patients. These findings expand the knowledge about HIV pathogenesis in untreated HIV-infected patients with different clinical outcomes.

## Figures and Tables

**Table 1:** Clinical and epidemiological characteristics of HIV infected patients and healthy donors.

Characteristics	Controls vs. All HIV Patients			HIV Groups of Patients			
	Control	All HIV ^(^*^)^	*p*-Value ^(a)^	LTNPs	MPs	RPs	*p*-Value ^(b)^
No.	113	667		183	334	150	
Male	93 (82.3%)	540 (81.4%)	0.829	115 (64.2%)	283 (84.7%)	142 (94.7%)	**<0.001**
Age (years)	42.0 (37.0; 49.0)	41.3 (35.0; 48.4)	0.427	48.7 (46.0; 51.7)	38.2 (33.2; 45.3)	38.3 (33.0; 43.8)	**<0.001**
Age of HIV diagnosis	–	34.3 (29.0; 40.4)	–	39.8 (34.3; 43.7)	31.8 (27.0; 38.4)	34.0 (29.6; 38.1)	**<0.001**
Year of HIV diagnosis	–	2006 (1999; 2008)	–	1993 (1990; 1997)	2006 (2004; 2008)	2009 (2007; 2010)	**<0.001**
HIV acquired	–		–				
IDU	–	166 (25.0%)	–	130 (72.6%)	29 (8.7%)	7 (4.7%)	**<0.001**
Homosexual	–	359 (54.1%)	–	13 (7.3%)	220 (65.9%)	126 (84.0%)	
Heterosexual	–	118 (17.8%)	–	27 (15.1%)	76 (22.8%)	15 (10.0%)	
Others	–	20 (3.0%)	–	9 (5.0%)	9 (2.7%)	2 (1.3%)	

*p*-values were calculated by Chi-square, Mann-Whitney and Kruskal-Wallis tests: ^(a)^ differences between control group and all HIV infected patients; ^(b)^ differences among HIV groups. Statistically significant differences are shown in bold. ^(^*^)^ Clinical and epidemiological data for three HIV-infected patients were not available. IDU, intravenous drug users; HIV, Human immunodeficiency virus; LTNPs, Long Term Non Progressors; MPs, Moderate Progressors; RPs, Rapid progressors.

**Table 2:** Characteristics of vitamin D receptor (*VDR)* polymorphisms in HIV infected patients and healthy donors.

SNPs	HWE	Control		HWE	All HIV		*p*-Value
rs11568820		*n* = 112			*n* = 665		
	0.616	CC	59.8%	0.501	CC	57.0%	0.800
		CT	33.9%		CT	35.3%	
		TT	6.3%		TT	7.7%	
rs1544410		*n* = 112			*n* = 666		
	0.233	CC	36.6%	0.835	CC	37.1%	0.477
		CT	42.9%		CT	46.8%	
		TT	20.5%		TT	16.1%	
rs2228570		*n* = 112			*n* = 664		
	0.474	CC	48.2%	0.812	CC	41.7%	0.435
		CT	40.2%		CT	44.9%	
		TT	11.6%		TT	13.4%	
rs4516035		*n* = 113			*n* = 666		
	0.149	TT	40.7%	0.895	TT	40.4%	0.202
		CT	40.7%		CT	46.8%	
		CC	18.6%		CC	12.8%	
rs7975232		*n* = 113			*n* = 662		
	0.294	AA	37.2%	0.862	AA	29.0%	0.217
		AC	43.4%		AC	48.9%	
		CC	19.5%		CC	22.1%	

*p*-values were calculated by Chi-squared test. VDR, vitamin D receptor; HWE, Hardy-Weinberg equilibrium; LTNPs, Long Term Non Progressors; MPs, Moderate Progressor; RPs, Rapid progressor.

## Appendix A

**Centers and investigators involved in CoRIS:**

**Executive committee**: Santiago Moreno, Julia del Amo, David Dalmau, Maria Luisa Navarro, Maria Isabel González, Jose Luis Blanco, Federico Garcia, Rafael Rubio, Jose Antonio Iribarren, Félix Gutiérrez, Francesc Vidal, Juan Berenguer, Juan González.

**Fieldwork, data management and analysis**: Julia Del Amo, Inma Jarrín, Belén Alejos, Victoria Hernando, Cristina Moreno, Carlos Iniesta, Luis Miguel Garcia Sousa, Nieves Sanz Perez.

**BioBanK HIV:** Hospital General Universitario Gregorio Marañón: M Ángeles Muñoz-Fernández, Isabel María García-Merino, Irene Consuegra Fernández, Coral Gómez Rico, Jorge Gallego de la Fuente, Paula Palau Concejo.

**Participating centres:**

**Hospital General Universitario de Alicante (Alicante):** Joaquín Portilla, Esperanza Merino, Sergio Reus, Vicente Boix, Livia Giner, Carmen Gadea, Irene Portilla, María Pampliega, Marcos Díez, Juan Carlos Rodríguez, José Sánchez-Payá.

**Hospital Universitario de Canarias (San Cristobal de la Laguna):** Juan Luis Gómez, Jehovana Hernández, María Remedios Alemán, María del Mar Alonso, María Inmaculada Hernández, Felicitas Díaz-Flores, Dácil García, Ricardo Pelazas, Ana López Lirola.

**Hospital Universitario Central de Asturias (Oviedo):** José Sanz Moreno, Alberto Arranz Caso, Cristina Hernández Gutiérrez, María Novella Mena.

**Hospital Universitario 12 de Octubre (Madrid):** Rafael Rubio, Federico Pulido, Otilia Bisbal, Asunción Hernando, Lourdes Domínguez, David Rial Crestelo, Laura Bermejo, Mireia Santacreu.

**Hospital Universitario de Donostia (Donostia-San Sebastián):** José Antonio Iribarren, Julio Arrizabalaga, María José Aramburu, Xabier Camino, Francisco Rodríguez-Arrondo, Miguel Ángel von Wichmann, Lidia Pascual Tomé, Miguel Ángel Goenaga, Mª Jesús Bustinduy, Harkaitz Azkune, Maialen Ibarguren, Aitziber Lizardi, Xabier Kortajarena.

**Hospital General Universitario De Elche (Elche):** Félix Gutiérrez, Mar Masiá, Sergio Padilla, Andrés Navarro, Fernando Montolio, Catalina Robledano, Joan Gregori Colomé, Araceli Adsuar, Rafael Pascual, Marta Fernández, Elena García., José Alberto García, Xavier Barber.

**Hospital General Universitario Gregorio Marañón (Madrid):** Juan Berenguer, Juan Carlos López Bernaldo de Quirós, Isabel Gutiérrez, Margarita Ramírez, Belén Padilla, Paloma Gijón, Teresa Aldamiz-Echevarría, Francisco Tejerina, Francisco José Parras, Pascual Balsalobre, Cristina Diez, Leire Pérez Latorre.

**Hospital Universitari de Tarragona Joan XXIII (Tarragona):** Francesc Vidal, Joaquín Peraire, Consuelo Viladés, Sergio Veloso, Montserrat Vargas, Miguel López-Dupla, Montserrat Olona, Anna Rull, Esther Rodríguez-Gallego, Verónica Alba.

**Hospital Universitario y Politécnico de La Fe (Valencia):** Marta Montero Alonso, José López Aldeguer, Marino Blanes Juliá, María Tasias Pitarch, Iván Castro Hernández, Eva Calabuig Muñoz, Sandra Cuéllar Tovar, Miguel Salavert Lletí, Juan Fernández Navarro.

**Hospital Universitario La Paz/IdiPAZ:** Juan González-garcia, Francisco Arnalich, José Ramón Arribas, Jose Ignacio Bernardino de la Serna, Juan Miguel Castro, Luis Escosa, Pedro Herranz, Victor Hontañón, Silvia García-Bujalance, Milagros García López-Hortelano, Alicia González-Baeza, Maria Luz Martín-Carbonero, Mario Mayoral, Maria Jose Mellado, Rafael Esteban Micán, Rocio Montejano, María Luisa Montes, Victoria Moreno, Ignacio Pérez-Valero, Berta Rodés, Talia Sainz, Elena Sendagorta, Natalia Stella Alcáriz, Eulalia Valencia.

**Hospital San Pedro Centro de Investigación Biomédica de La Rioja (CIBIR) (Logroño):** José Ramón Blanco, José Antonio Oteo, Valvanera Ibarra, Luis Metola, Mercedes Sanz, Laura Pérez-Martínez.

**Hospital Universitari MutuaTerrassa (Terrasa):** David Dalmau, Angels Jaén, Montse Sanmartí, Mireia Cairó, Javier Martinez-Lacasa, Pablo Velli, Roser Font, Mariona Xercavins, Noemí Alonso.

**Complejo Hospitalario de Navarra (Pamplona)** María Rivero, Jesús Repáraz, María Gracia Ruiz de Alda, María Teresa de León Cano, Beatriz Pierola Ruiz de Galarreta.

**Corporació Sanitària Parc Taulí (Sabadell):** Ferrán Segura, María José Amengual, Gemma Navarro, Montserrat Sala, Manuel Cervantes, Valentín Pineda, Sonia Calzado, Marta Navarro.

**Hospital Universitario de La Princesa (Madrid):** Ignacio de los Santos, Jesús Sanz Sanz, Ana Salas Aparicio, Cristina Sarriá Cepeda, Lucio Garcia-Fraile Fraile, Enrique Martín Gayo.

**Hospital Universitario Ramón y Cajal (Madrid):** Santiago Moreno, José Luis Casado, Fernando Dronda, Ana Moreno, María Jesús Pérez Elías, Cristina Gómez Ayerbe, Carolina Gutiérrez, Nadia Madrid, Santos del Campo Terrón, Paloma Martí, Uxua Ansa, Sergio Serrano, María Jesús Vivancos.

**Hospital General Universitario Reina Sofía (Murcia)** Enrique Bernal, Alfredo Cano, Antonia Alcaraz García, Joaquín Bravo Urbieta, Ángeles Muñoz, Maria Jose Alcaraz, Maria del Carmen Villalba.

**Hospital Nuevo San Cecilio (Granada):** Federico García, José Hernández, Alejandro Peña, Leopoldo Muñoz, Paz Casas, Marta Alvarez, Natalia Chueca, David Vinuesa, Clara Martinez-Montes.

**Centro Sanitario Sandoval (Madrid):** Jorge Del Romero, Carmen Rodríguez, Teresa Puerta, Juan Carlos Carrió, Mar Vera, Juan Ballesteros, Oskar Ayerdi.

**Hospital Universitario Son Espases (Palma de Mallorca):** Melchor Riera, María Peñaranda, María Leyes, Mª Angels Ribas, Antoni A Campins, Carmen Vidal, Francisco Fanjul, Javier Murillas, Francisco Homar.

**Hospital Universitario Virgen de la Victoria (Málaga):** Jesús Santos, Crisitina Gómez Ayerbe, Isabel Viciana, Rosario Palacios, Carmen María González.

**Hospital Universitario Virgen del Rocío (Sevilla):** Pompeyo Viciana, Nuria Espinosa, Luis Fernando López-Cortés.

**Hospital Universitario de Bellvitge (Hospitalet de Llobregat):** Daniel Podzamczer, Elena Ferrer, Arkaitz Imaz, Juan Tiraboschi, Ana Silva, María Saumoy.

**Hospital Costa del Sol (Marbella):** Julián Olalla, Alfonso del Arco, Javier de la torre, José Luis Prada, José María García de Lomas Guerrero, Javier Pérez Stachowski.

**Hospital General Universitario Santa Lucía (Cartagena):** Onofre Juan Martínez, Francisco Jesús Vera, Lorena Martínez, Josefina García, Begoña Alcaraz, Amaya Jimeno.

Complejo Hospitalario Universitario a Coruña (Chuac) (A Coruña): Angeles Castro Iglesias, Berta Pernas Souto, Alvaro Mena de Cea.

**Hospital Universitario Virgen de la Arrixaca (El Palmar):** Carlos Galera, Helena Albendin, Aurora Pérez, Asunción Iborra, Antonio Moreno, Maria Angustias Merlos, Asunción Vidal.

**Hospital Universitario Infanta Sofia (San Sebastian de los Reyes):** Inés Suárez-García, Eduardo Malmierca, Patricia González-Ruano, Dolores Martín Rodrigo.

**Complejo Hospitalario de Jaén (Jaén)** Mohamed Omar Mohamed-Balghata, María Amparo Gómez Vidal.

**Hospital Clínico San Carlos (Madrid):** Vicente Estrada Pérez, Maria Jesus Téllez Molina, Jorge Vergas García, Juncal Pérez-Somarriba Moreno.

**Hospital Universitario Fundación Jiménez Díaz (Madrid):** Miguel Górgolas, Alfonso Cabello., Beatriz Álvarez., Laura Prieto.

**Hospital Universitario Príncipe de Asturias (Alcalá de Henares):** José Sanz Moreno, Alberto Arranz Caso, Cristina Hernández Gutiérrez, María Novella Mena.

**Hospital Clínico Universitario de Valencia (València):** María José Galindo Puerto, Ramón Fernando Vilalta, Ana Ferrer Ribera.

**Hospital Reina Sofía (Córdoba):** Antonio Rivero Román, Maria Teresa Brieva Herrero, Antonio Rivero Juárez, Pedro López López, Isabel Machuca Sánchez, José Peña Martínez.

**Hospital Universitario Severo Ochoa (Leganés)**: Miguel Cervero Jiménez, Rafael Torres Perea, Juan José Jusdado Ruiz-Capillas.

**Nuestra Señora de Valme:** Juan A Pineda.

## Appendix B

**Centers involved in Long Term Non-Progressors (LTNP) chort:**

Centro Sanitario Sandoval—Madrid
Hospital 12 de Octubre—Madrid
Hospital Arnau de Vilanova—Lleida
Hospital Asturias
Hospital Bellvitge—Barcelona
Hospital Castellón
Hospital Clínic—Barcelona
Hospital Donostia—San Sebastián
Hospital Elche—Alicante
Hospital Germans Trias i Pujol—Badalona
Hospital Gregorio Marañón—Madrid
Hospital Joan XXIII—Tarragona
Hospital La Fe—Valencia
Hospital La Paz/Carlos III—Madrid
Hospital La Princesa—Madrid
Hospital Navarra—Pamplona
Hospital Parc Taulí- Sabadell
Hospital Ramón y Cajal—Madrid
Hospital San Cecilio—Granada
Hospital San Pedro—Logroño
Hospital Son Dureta—Mallorca
Hospital Virgen del Rocío—Sevilla

## References

[1] Munoz A., Sabin C.A., Phillips A.N. (1997). The incubation period of AIDS. AIDS.

[2] Pantaleo G., Fauci A.S. (1996). Immunopathogenesis of HIV infection. Ann. Rev. Microbiol..

[3] Gurdasani D., Iles L., Dillon D.G., Young E.H., Olson A.D., Naranbhai V., Fidler S., Gkrania-Klotsas E., Post F.A., Kellam P. (2014). A systematic review of definitions of extreme phenotypes of HIV control and progression. AIDS.

[4] Estes J.D., LeGrand R., Petrovas C. (2018). Visualizing the immune system: Providing key insights into HIV/SIV infections. Front. Immunol..

[5] Biasin M., De Luca M., Gnudi F., Clerici M. (2013). The genetic basis of resistance to HIV infection and disease progression. Expert Rev. Clin. Immunol..

[6] Autier P., Boniol M., Pizot C., Mullie P. (2014). Vitamin D status and ill health: A systematic review. Lancet Diabetes Endocrinol..

[7] Jimenez-Sousa M.A., Martinez I., Medrano L.M., Fernandez-Rodriguez A., Resino S. (2018). Vitamin D in human immunodeficiency virus infection: Influence on immunity and disease. Front. Immunol..

[8] Lucas R.M., Gorman S., Geldenhuys S., Hart P.H. (2014). Vitamin D and immunity. F1000Prime Rep..

[9] Mansueto P., Seidita A., Vitale G., Gangemi S., Iaria C., Cascio A. (2015). Vitamin D deficiency in HIV infection: Not only a bone disorder. BioMed Res. Int..

[10] Gois P.H.F., Ferreira D., Olenski S., Seguro A.C. (2017). Vitamin D and infectious diseases: Simple bystander or contributing factor?. Nutrients.

[11] Hossein-nezhad A., Holick M.F. (2013). Vitamin D for health: A global perspective. Mayo Clin. Proc..

[12] Fang Y., van Meurs J.B., d’Alesio A., Jhamai M., Zhao H., Rivadeneira F., Hofman A., van Leeuwen J.P., Jehan F., Pols H.A. (2005). Promoter and 3’-untranslated-region haplotypes in the vitamin D receptor gene predispose to osteoporotic fracture: The rotterdam study. Am. J. Hum. Genet..

[13] Nejentsev S., Godfrey L., Snook H., Rance H., Nutland S., Walker N.M., Lam A.C., Guja C., Ionescu-Tirgoviste C., Undlien D.E. (2004). Comparative high-resolution analysis of linkage disequilibrium and tag single nucleotide polymorphisms between populations in the vitamin D receptor gene. Hum. Mol. Genet..

[14] Jolliffe D.A., Walton R.T., Griffiths C.J., Martineau A.R. (2016). Single nucleotide polymorphisms in the vitamin D pathway associating with circulating concentrations of vitamin D metabolites and non-skeletal health outcomes: Review of genetic association studies. J. Steroid Biochem. Mol. Biol..

[15] Barber Y., Rubio C., Fernandez E., Rubio M., Fibla J. (2001). Host genetic background at CCR5 chemokine receptor and vitamin D receptor loci and human immunodeficiency virus (HIV) type 1 disease progression among HIV-seropositive injection drug users. J. Infect. Dis..

[16] Laplana M., Sanchez-de-la-Torre M., Puig T., Caruz A., Fibla J. (2014). Vitamin-D pathway genes and HIV-1 disease progression in injection drug users. Gene.

[17] Nieto G., Barber Y., Rubio M.C., Rubio M., Fibla J. (2004). Association between AIDS disease progression rates and the Fok-I polymorphism of the *VDR* gene in a cohort of HIV-1 seropositive patients. J. Steroid Biochem. Mol. Biol..

[18] Torres C., Sanchez de la Torre M., Garcia-Moruja C., Carrero A.J., Trujillo Mdel M., Fibla J., Caruz A. (2010). Immunophenotype of vitamin D receptor polymorphism associated to risk of HIV-1 infection and rate of disease progression. Curr. HIV Res..

[19] Garcia-Merino I., de Las Cuevas N., Jimenez J.L., Gallego J., Gomez C., Prieto C., Serramia M.J., Lorente R., Munoz-Fernandez M.A. (2009). The Spanish HIV BioBank: A model of cooperative HIV research. Retrovirology.

[20] Sobrino-Vegas P., Gutierrez F., Berenguer J., Labarga P., Garcia F., Alejos-Ferreras B., Munoz M.A., Moreno S., del Amo J. (2011). The Cohort of the Spanish HIV Research Network (CoRIS) and its associated biobank; organizational issues, main findings and losses to follow-up. Enferm. Infec. Microbiol. Clin..

[21] Guzman-Fulgencio M., Jimenez J.L., Garcia-Alvarez M., Bellon J.M., Fernandez-Rodriguez A., Campos Y., Rodriguez C., Gonzalez-Garcia J., Riera M., Viciana P. (2013). Mitochondrial haplogroups are associated with clinical pattern of AIDS progression in HIV-infected patients. J. Acquir. Immune Defic. Syndr..

[22] Guzman-Fulgencio M., Jimenez J.L., Jimenez-Sousa M.A., Bellon J.M., Garcia-Alvarez M., Soriano V., Gijon-Vidaurreta P., Bernal-Morell E., Viciana P., Munoz-Fernandez M.A. (2014). ACSM4 polymorphisms are associated with rapid AIDS progression in HIV-infected patients. J. Acquir. Immune Defic. Syndr..

[23] Alagarasu K., Selvaraj P., Swaminathan S., Narendran G., Narayanan P.R. (2009). 5′ regulatory and 3′ untranslated region polymorphisms of vitamin D receptor gene in south Indian HIV and HIV-TB patients. J. Clin. Immunol..

[24] De la Torre M.S., Torres C., Nieto G., Vergara S., Carrero A.J., Macias J., Pineda J.A., Caruz A., Fibla J. (2008). Vitamin D receptor gene haplotypes and susceptibility to HIV-1 infection in injection drug users. J. Infect. Dis..

[25] Aguilar-Jimenez W., Villegas-Ospina S., Gonzalez S., Zapata W., Saulle I., Garziano M., Biasin M., Clerici M., Rugeles M.T. (2016). Precursor forms of vitamin D reduce HIV-1 infection in vitro. J. Acquir. Immune Defic. Syndr..

[26] Nunnari G., Fagone P., Lazzara F., Longo A., Cambria D., Di Stefano G., Palumbo M., Malaguarnera L., Di Rosa M. (2016). Vitamin D3 inhibits TNFα-induced latent HIV reactivation in J-LAT cells. Mol. Cell. Biochem..

[27] Mendes M.M., Darling A.L., Hart K.H., Morse S., Murphy R.J., Lanham-New S.A. (2019). Impact of high latitude, urban living and ethnicity on 25-hydroxyvitamin D status: A need for multidisciplinary action?. J. Steroid Biochem. Mol. Biol..

[28] Gross C., Eccleshall T.R., Malloy P.J., Villa M.L., Marcus R., Feldman D. (1996). The presence of a polymorphism at the translation initiation site of the vitamin D receptor gene is associated with low bone mineral density in postmenopausal Mexican-American women. J. Bone Min. Res..

[29] Laplana M., Royo J.L., Fibla J. (2018). Vitamin D Receptor polymorphisms and risk of enveloped virus infection: A meta-analysis. Gene.

[30] Ogunkolade B.W., Boucher B.J., Prahl J.M., Bustin S.A., Burrin J.M., Noonan K., North B.V., Mannan N., McDermott M.F., DeLuca H.F. (2002). Vitamin D receptor (VDR) mRNA and VDR protein levels in relation to vitamin D status, insulin secretory capacity, and VDR genotype in Bangladeshi Asians. Diabetes.

[31] Kholghi Oskooei V., Geranpayeh L., Omrani M.D., Ghafouri-Fard S. (2018). Assessment of functional variants and expression of long noncoding RNAs in vitamin D receptor signaling in breast cancer. Cancer Manag. Res..

[32] Moran-Auth Y., Penna-Martinez M., Shoghi F., Ramos-Lopez E., Badenhoop K. (2013). Vitamin D status and gene transcription in immune cells. J. Steroid Biochem. Mol. Biol..

[33] Vanessa O.N., Asani F.F., Jeffery T.J., Saccone D.S., Bornman L. (2013). Vitamin D receptor gene expression and function in a south African population: Ethnicity, vitamin D and FokI. PLoS ONE.

